# Antimicrobial resistance of pathogens causing nosocomial bloodstream infection in Hubei Province, China, from 2014 to 2016: a multicenter retrospective study

**DOI:** 10.1186/s12889-018-6013-5

**Published:** 2018-09-15

**Authors:** Lei Tian, Ziyong Sun, Zhen Zhang

**Affiliations:** 1 Department of Clinical Laboratory, Tongji Hospital, Tongji Medical College, Huazhong University of Science and Technology, Wuhan, Hubei Province China; 2 Department of Pharmacy, Tongji Hospital, Tongji Medical College, Huazhong University of Science and Technology, Wuhan, Hubei Province China

**Keywords:** Nosocomial bloodstream infection, Antimicrobial resistance, MRSA, ESBL

## Abstract

**Background:**

Data on the pathogens responsible for nosocomial bloodstream infection (BSI) and their antimicrobial resistance (AMR) in Hubei province are limited. This study was conducted to determine the major pathogens causing BSI and to characterize their AMR.

**Methods:**

Data from the China Antimicrobial Resistance Surveillance System (CARSS) from 2014 to 2016 were analyzed retrospectively.

**Results:**

*Escherichia coli, Staphylococcus aureus* and *Klebsiella pneumoniae* were the most common pathogens responsible for nosocomial BSI. Individuals aged 0–5 years and ≥ 40 years old were the major demographics at risk of infection by *E. coli, K. pneumoniae, Pseudomonas aeruginosa, Acinetobacter baumannii* and *Enterobacter cloacae*, while individuals aged 0–5 years were the major demographic at risk of infection by *S. aureus, Enterococcus faecalis, E. faecium, Streptococcus pneumoniae* and *Stenotrophomonas maltophilia*. The frequencies of *E. coli* and *K. pneumoniae* isolates resistant to cefotaxime were 59.1% and 24.3%, respectively, and the frequencies of resistant isolates to ceftazidime were 42.9% and 27.2%, respectively. From 2014 to 2016, the frequency of extended-spectrum β-lactamase (ESBL)-positive *E. coli* declined from 29.07 to 24.5%, and the frequency of ESBL-positive *K. pneumoniae* declined from 18.64 to 12.33%. The frequency of carbapenem-resistant (CR) *E. coli* was below 0.5%, but 1–10% of *K. pneumoniae* isolates were CR.

**Conclusions:**

The emergence of methicillin-resistant *S. aureus* and the expansion of ESBL and fluoroquinolone resistance among Gram-negative *Enterobacteriaceae* increased AMR severity. Carbapenemase-producing *K. pneumoniae* isolates responsible for nosocomial BSI increased year over year and effective infection control measures should be taken to prevent them from spreading.

## Background

Bloodstream infection (BSI) is very common and is associated with high morbidity and mortality worldwide. The etiology of BSI and the antimicrobial resistance (AMR) of the pathogens causing it differ significantly between developed and developing countries [1]. Data from the Finnish Hospital Infection Program during 1999–2001 and 2005–2010 demonstrated that the most common pathogens responsible for BSI were coagulase-negative *Staphylococcus*, *S. aureus* and *Escherichia coli* [2]. By contrast, BSI surveillance in Malawi from 1998 to 2016 indicated that non-typhoidal *Salmonella*, *S. enterica Typhi* and *Streptococcus pneumoniae* were the most common causative agents [3]. In China, the pathogens responsible for BSI vary from region to region. A study of the clinical features of nosocomial BSI in neonates from two prefectures, Henan and Chongqing, indicated that *E. coli* was the most prevalent BSI pathogen in Henan while *Klebsiella pneumoniae* was the predominant BSI pathogen in Chongqing [4]. Reports on the causes of BSI in Hubei province have been limited.

**Fig. 1:**
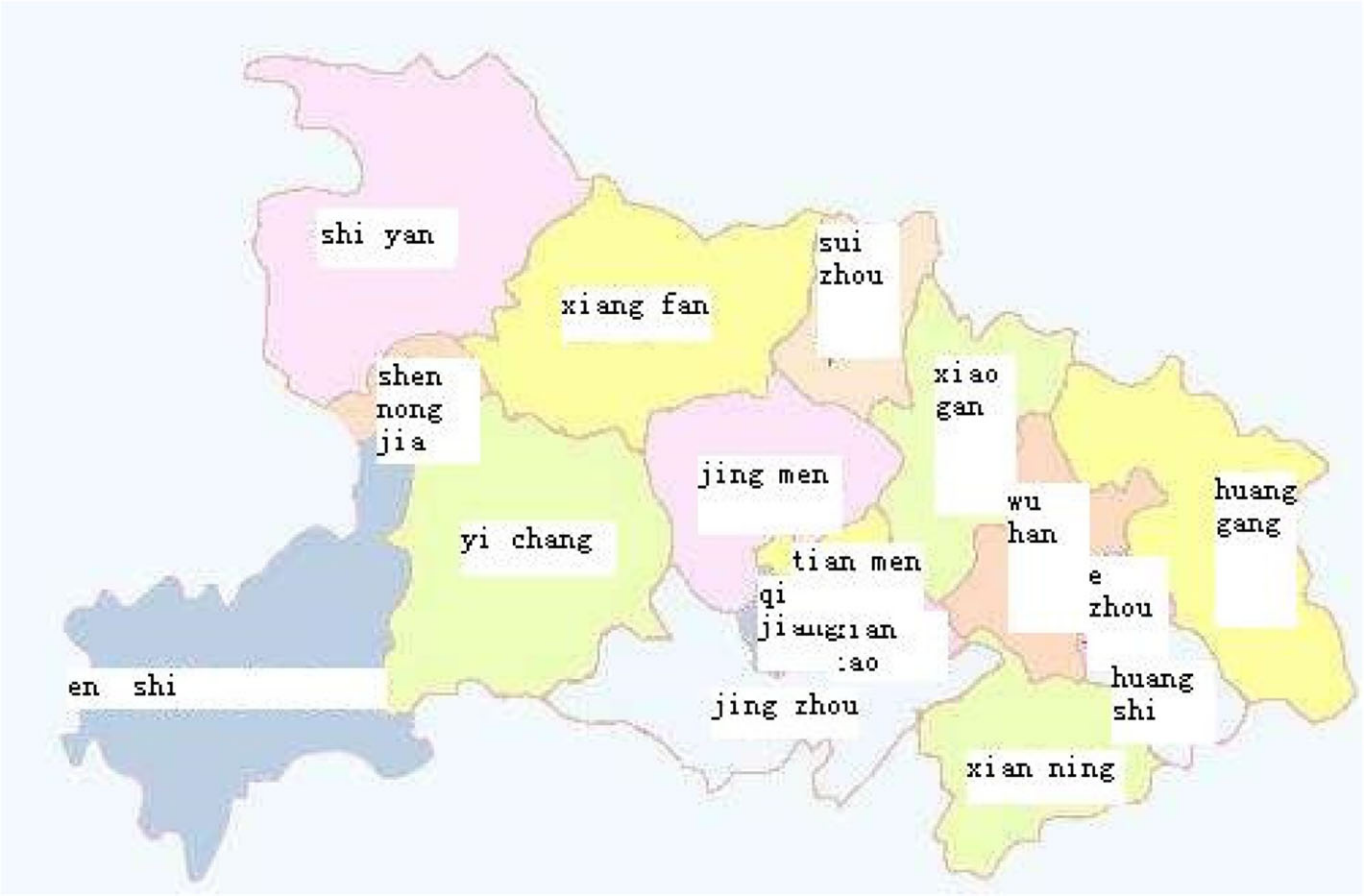
Location distribution of the members of the monitoring network

Surveillance and analysis of AMR are crucial in preventing BSI. The Hubei Province bacterial resistance surveillance network was founded in 1998 and includes surveillance data on hospital-acquired and community-acquired infections. By the end of 2016, a total of 11 secondary care hospitals and 41 tertiary care hospitals had joined the network, covering all prefecture-level cities in Hubei Province (Fig. 1). Here, in order to provide a basis for empirical treatment and policy making for the Department of Health Administration, we reported BSI surveillance data over the last three years, from 2014 to 2016.

## Methods

### Study design and data collection

We undertook a retrospective surveillance study of nosocomial BSI in Hubei province. The data were derived from the China Antimicrobial Resistance Surveillance System (CARSS). Hospital-acquired, nosocomial, and health care-associated infections were defined according to the World Health Organization (WHO) as infections occurring in a patient residing in a hospital or another health care facility, in whom the infection was not present nor incubating at the time of admission [5]. An infection occurring 48 h or later after admission to hospital was considered nosocomial [5]. An infection occurring within 48 h of admission to hospital or not associated with healthcare interventions within the previous 12 months was defined as community-acquired [6]. If the isolates were identified as coagulase-negative *Staphylococcus, Corynebacterium, Bacillus*, *Propionibacterium* or other potential skin contaminants, two or more separate blood cultures were required for diagnosis [7].

### Strain identification and antibiotic sensitivity testing

Blood samples (8–10 mL for adult patients and 2–5 mL for pediatric patients) were collected into special blood culture bottles. Various automated blood culture instruments were applied. Manual biochemical tests, automatic bacteria identification instruments and/or an IVD-MALDI Biotyper (Bruker, Germany) were used for identification of bacterial species. The Kirby-Bauer method and/or the minimum inhibitory concentration (MIC) method were used for antimicrobial susceptibility testing according to the Clinical and Laboratory Standards Institute (CLSI) guidelines. The results of antimicrobial sensitivity tests were interpreted according to the CLSI 2016 standards [8]. Isolation and identification of strains and antibiotic sensitivity testing were performed by members of the bacterial resistance monitoring network. Each member of the monitoring network reported data regarding bacterial identification and antibiotic sensitivity to the CARSS each quarter.

### Statistical analysis

Antibiotic susceptibility data was analyzed using WHONET 5.6 software. Only the first isolate of a given species from a patient was analyzed according to CLSI M-39 [9]. When antibiotic resistance tests were carried out by the Kirby-Bauer method, the MIC method and the E-test method simultaneously, the results of E-tests were preferred, followed by the MIC method and the Kirby-Bauer method.

## Results

### Distribution of common pathogens responsible for BSI

**Fig. 2:**
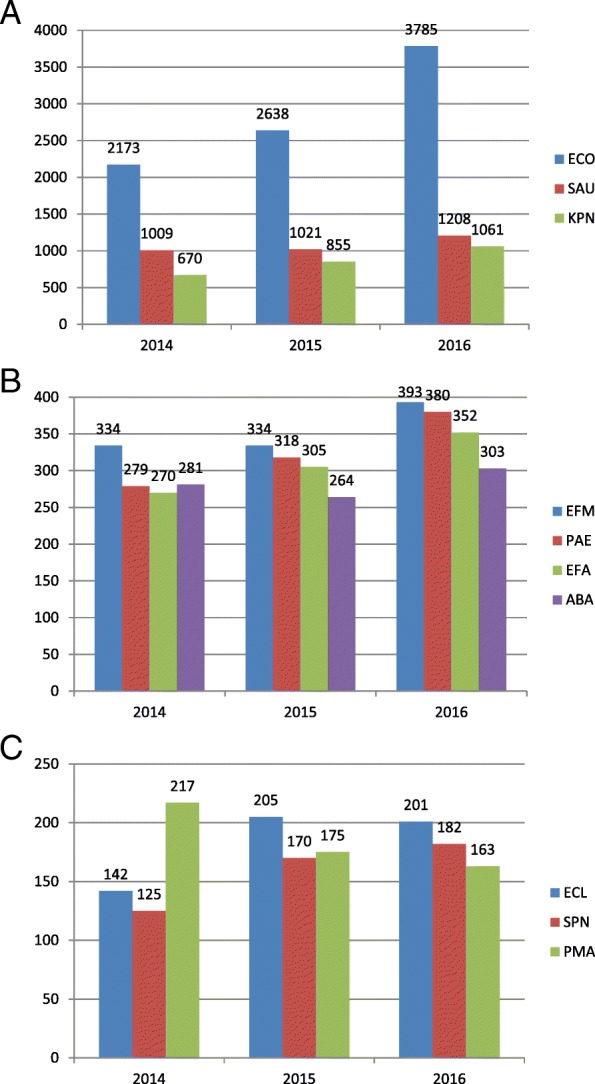
Pathogens in bloodstream infection, 2014~ 2016. **a** Pathogens isolated at high frequency (*N* > 500/year). **b** Pathogens isolated at intermediate frequency (250/year<*N* < 500/year). **c** Pathogens isolated at low frequency (100/year<*N* < 250/year)

In this investigation, only strains isolated more than 100 times per year were analyzed. *E. coli* was the most common pathogen responsible for nosocomial BSI during 2014–2016, followed by *S. aureus* and *K. pneumonia*. Each of these pathogens was isolated more than 500 times per year (Fig. 2a). *Enterococcus faecium, E. faecalis, Pseudomonas aeruginosa* and *Acinetobacter Baumannii* isolated were each isolated between 250 and 500 times per year (Fig. 2b). *Enterobacter cloacae*, *S. pneumoniae* and *Stenotrophomonas maltophilia* were each isolated between 100 and 250 times per year (Fig. 2c).

### Age demographics affected by BSI

**Fig. 3:**
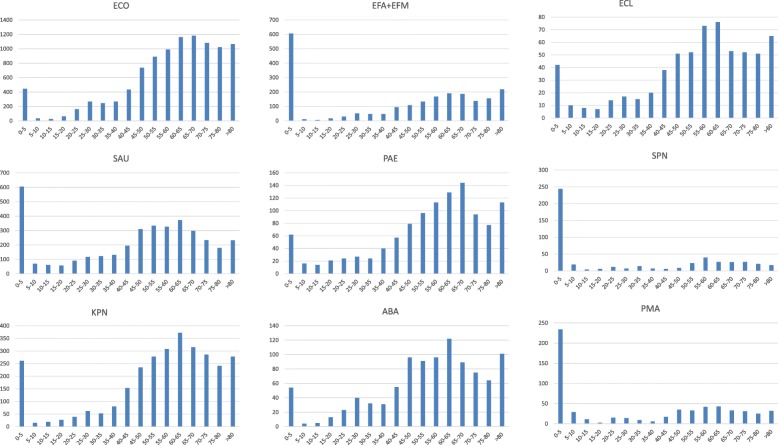
The main pathogens isolated from bloodstream infection stratified by age

Individuals aged 0–5 years and ≥ 40 years old were the major demographics at risk of infection by *E. coli, K. pneumoniae, P. aeruginosa, A.* baumannii and *E. cloacae*, while individuals aged 0–5 years were the major demographic at risk of infection by *S. aureus, E. faecalis, E. faecium, S. pneumoniae* and *S. maltophilia* (Fig. 3).

### Seasonal distribution of BSI pathogens

**Fig. 4:**
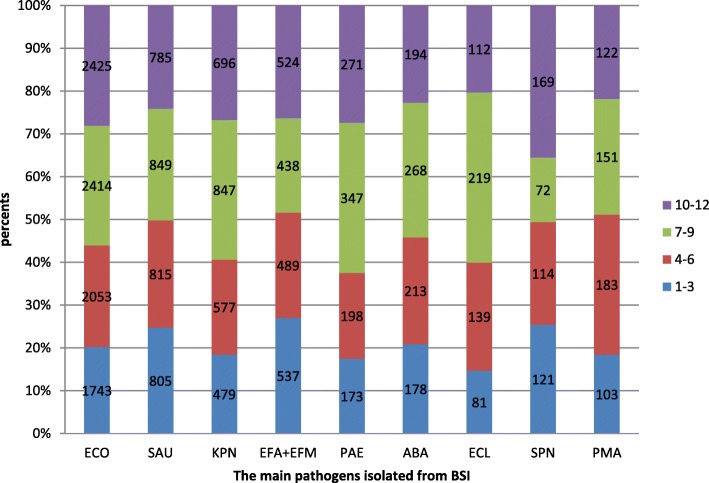
The distribution proportion in the four seasons of the main pathogens isolated from BSI

Most *E. coli* isolates were identified from October to December (10–12) and from July to September (7–9), with the lowest number identified from January to March (1–3). Isolates of *S. aureus, E. faecalis* and *E. faecium* were distributed roughly evenly across all four seasons. The highest number of *K. pneumoniae, P. aeruginosa, A. baumannii* and *E. cloacae* isolates identified in a quarter was 7–9 and the lowest number of these species was 1–3. Most *S. maltophilia* isolates were identified from April to June (4–6) and the lowest number observed in a quarter was 1–3 (Fig. 4).

### Antimicrobial susceptibility

**Fig. 5:**
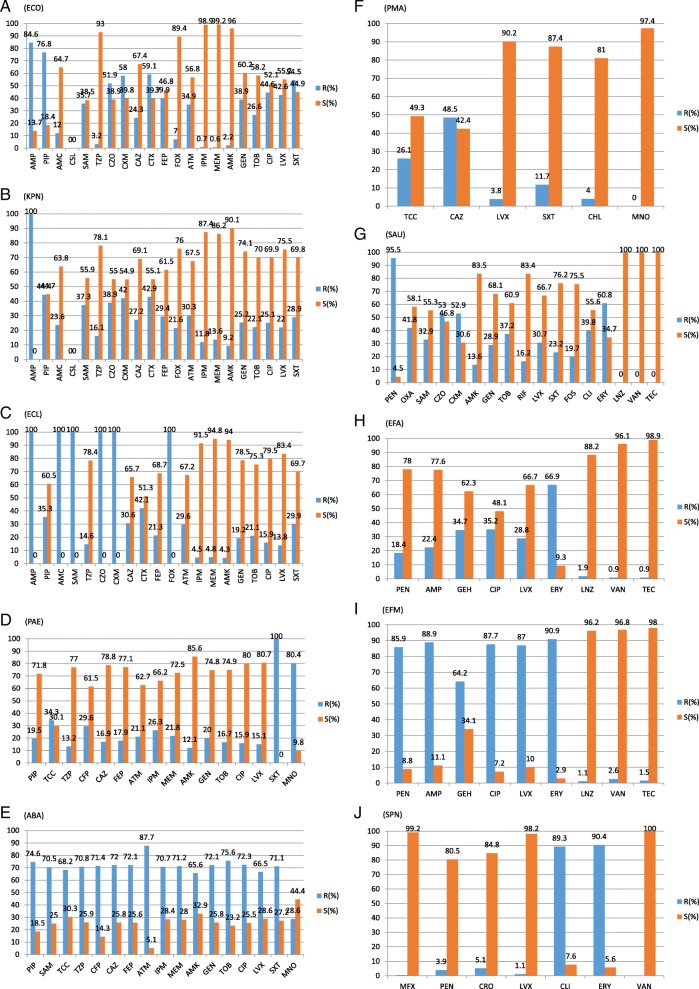
Susceptibility rates and resistance rates to common antibiotics. **a** (*Escherichia coli*). **b** (*Klebsiella pneumoniae*). **c** (*Enterobacter cloacae*). **d** (Pseudomonas aeruginosa). **e** (Acinetobacter Bauman). **f** (Stenotrophomonas maltophilia). **g **(Staphylococcus aureus). **h** (Enterococcus faecalis). **i** (Enterococcus faecium). **j** (Streptococcus pneumonia)

For *E. coli* isolates, resistance rates to ampicillin (AMP), piperacillin (PIP), cefazolin (CZO), cefuroxime (CXM), cefotaxime (CTX) and trimethoprim sulfamethoxazole (SXT) were > 50%, but more than 89% of isolates were sensitive to imipenem (IPM), meropenem (MEM), piperacillin-tazobactam (TZP), amikacin (AMK) and cefoxitin (FOX) (Fig. 5a). *K. pneumoniae* isolates were naturally resistant to AMP, and resistance rates to all other antibiotics except for PIP were > 50% (Fig. 5b). *E. cloacae* isolates were naturally resistant to AMP, PIP, CZO, CXM, FOX and ampicillin-sulbactam (SAM), but > 50% of isolates were sensitive to the other antibiotics (Fig. 5c). *P. aeruginosa* isolates were naturally resistant to SXT, and the resistance rate to minocycline (MNO) was 80.4%. However, > 60% of isolates were sensitive the other antibiotics except for ticarcillin-clavulanate Acid (TCC) (Fig. 5d). More than 65% of *A. baumannii* isolates were resistant to common antibiotics except for MNO (Fig. 5e). Sensitivity rates of *S. maltophilia* isolates to SXT, MNO, levofloxacin (LVX) and chloramphenicol (CHL) exceeded 80% (Fig. 5f). More than 50% of *S. aureus* isolates were resistant to penicillin (PEN), erythromycin (ERY), CZO and CXM (Fig. 5g). More than 60% of *E. faecalis* isolates were sensitive to common antibiotics except for ERY and ciprofloxacin (CIP) (Fig. 5h). For *E. faecium* isolates, sensitivity rates to vancomycin (VAN), teicoplanin (TEC) and linezolid (LNZ) exceeded 95%, but resistance rates to other antibiotics exceeded 60% (Fig. 5i). For *S. pneumoniae* isolates, resistance rates to ERY and clindamycin (CLI) exceeded 89%, but sensitivity rates to other antibiotics exceeded 80% (Fig. 5j).

### Trends of multi-drug resistant (MDR) strains

**Fig. 6:**
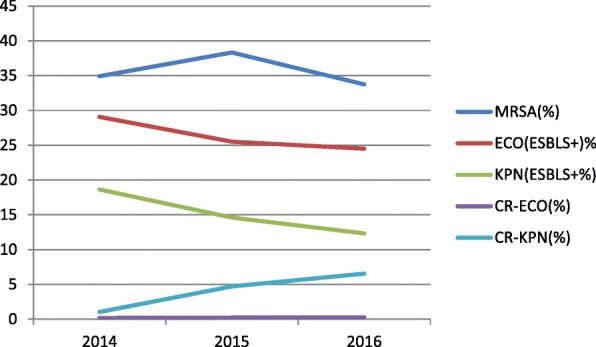
Trends of common multi-drug resistant strains from 2014 to 2016

The frequency of methicillin-resistant *S. aureus* (MRSA), extended-spectrum β-lactamase (ESBL)-positive *E. coli* and *K. pneumoniae*, and carbapenem-resistant (CR) *E. coli* and *K. pneumoniae* were analyzed during 2014–2016. The proportion of MRSA isolates ranged from 30 to 40%, ESBL-positive *E. coli* isolates ranged from 20 to 30%, and ESBL-positive *K. pneumoniae* isolates ranged from 10 to 20%. The frequency of CR-*E. coli* was below 0.5%, but CR-*K. pneumoniae* isolates were detected at a frequency of 1–10% (Fig. 6).

## Discussion

Our surveillance data indicated *E. coli, S. aureus* and *K. pneumoniae* were the most common pathogens responsible for nosocomial BSI in Hubei province from 2014 to 2016. For common gram-positive bacteria and *S. maltophilia*, individuals < 5 years of age were the major demographic at risk of nosocomial BSI. By contrast, for common gram-negative bacteria except *S. maltophilia*, individuals < 5 years of age and ≥ 40 years of age were the main demographics at risk of BSI. Resistance of *E. coli* isolates to common antibiotics was more severe than *K. pneumoniae* isolates, especially to third-generation cephalosporins and fluoroquinolones. ESBL production by *E. coli* isolates was more common than in *K. pneumoniae* isolates. Most *P. aeruginosa* isolates (> 60%) were sensitive to antibiotics, while most *A. baumannii* isolates (> 60%) were antibiotic-resistant.

*E. coli, S. aureus* and *K. pneumoniae* were the most common pathogens responsible for BSI according to our surveillance data. These results differed from reports in European and African populations, in which coagulase-negative *Staphylococcus* and non-typhoidal *Salmonella* were the predominant causative agents of BSI (in Finland and Malawi, respectively) [2, 3]. This may be due to the distinct social, economic and environmental factors in these regions. The spectrum of pathogens responsible for BSI is highly variable both geographically and temporally [10]. Timely analysis and reporting of local AMR resistant isolates is important for infection control.

The most common pathogens responsible for BSI are different in people of different ages [3]. Our study found that individuals aged 0–5 years old were an important demographic at high risk of BSI. It has been well established that children under 5 years of age are susceptible to infection by *Salmonella* and other intestinal pathogens [11]. Our findings indicated that children under 5 years of age were also the main demographic susceptible to BSI. For Gram-positive bacteria (*S. aureus, E. faecalis, E. faecium,* and *S. pneumoniae*) and *S. maltophilia*, individuals under 5 years of age were the major demographic at risk, while individuals above 40 years of age or under 5 years of age were the primary population at risk of infection by Gram-negative bacteria (*E. coli, K. pneumoniae, E cloacae, P. aeruginosa* and *A. baumanni*). Our findings indicated that the incidence of *E. coli* and *K. pneumoniae* BSIs was highest in elderly patients older than 40 years, consistent with global data [12]. The prolongation of human life and the aging of populations in society maybe have something to do with increasing rates of BSI caused by *E. coli* and *K. pneumoniae*.

It was reported that BSI by *Bacillus cereus* had a seasonal tendency with increased risk of infection during the summer [13]. Our data showed that *S. pneumoniae* was more often isolated in winter while *K. pneumoniae, P. aeruginosa, A. baumanni, and E. cloacae* were most prevalent in autumn. No obvious seasonal trend was observed for *S. aureus, E. faecalis* and *E. faecium.* Whether BSIs have seasonal trends like food-borne diseases is worth further study.

The frequency of ESBL-producing *E. coli* and *K. pneumoniae* isolates has been increasing worldwide, resulting in prolonged hospital stays, increased costs, reduced rates of clinical and microbiological responses, and poor outcomes for patients and healthcare systems [14–20]. Previous studies have analyzed the relationship between mortality rate and ESBL-producing pathogens and observed an association with KP-BSIs, although this finding remains controversial [16, 21–23]. The frequency of ESBL-producing *E. coli* and *K. pneumoniae* isolates decreased year by year from 2014 to 2016 according to our surveillance data. This finding may be related to the strategies implemented by the USA CDC for controlling the transmission of MDR bacteria such as quarantine of patients with MDR infections, strict hand disinfection by medical staff, routine disinfection of medical devices and limited use of specific antibiotics. For *E. coli,* resistances rate to the third-generation cephalosporins CTX and CAZ were 59.1% and 24.3%, respectively; ESBL may be the main cause of resistance, especially for bla(CTX-M) isolates. The molecular epidemiology of *E. coli* isolates causing BSI in Shanghai during 2011–2013 demonstrated that CTX-14, CTX-55 and CTX-15 were the most common β-lactamases [24]. By contrast, bla(SHV) and bla(CTX-M) were the most common β-lactamases in *K. pneumoniae* isolates [25]. MDR and the increase in infections caused by Gram-negative bacilli producing ESBL have contributed to intensive use of carbapenems [26]. Data from the European Survey on Carbapenemase-Producing Enterobacteriaceae in Europe (EuSCAPE) indicated that the incidence of carbapenemase-producing (CP) *E. coli* and *K. pneumoniae* (CP-E/K) isolates in hospitals increased from 0.124 per 1000 admissions in 2013 to 0.223 per 1000 admissions in 2014 [27]. Our data showed that CP-E/K isolates are a growing risk, especially CP-*K. pneumoniae*.

In our surveillance data, *S. aureus* was the most frequently observed Gram-positive pathogen responsible for BSI. MRSA is a worldwide issue associated with significant morbidity and mortality [28]. Our data showed that the frequency of methicillin resistance among *S. aureus* isolates was 30–40%,consistent with a study conducted in 26 Hong Kong public hospitals between January 2010 and December 2012 [29], In the United States and Europe, multiple infection control approaches have been tested to control the spread of MRSA. These approaches included strict contact precautions including single rooms for MRSA-colonized or -infected patients, targeted admission screening for high-risk patients and healthcare workers at risk for infection, molecular typing of all MRSA strains, and routine decolonization of MRSA carriers including healthcare workers [28, 30, 31]. Measures to control MRSA transmission should be carried out strictly according to the principles of infection control.

This study had several limitations. In our surveillance data, only nosocomial BSI was analyzed and community-acquired BSI was not included. In Hubei province, the characteristics of community-acquired BSI are mostly unknown. The molecular mechanisms of MDR, especially ESBLs and carbapenemases, have not been tested. Molecular epidemiological studies need be carried out in the future.

## Conclusions

Our findings indicated that *E. coli*, *S. aureus* and *K. pneumoniae* were the most common pathogens responsible for nosocomial BSI in Hubei province. Individuals aged 0–5 years were the major demographic at risk of infection by *S. aureus*, *E. faecalis*, *E. faecium*, *S. pneumoniae* and *S. maltophilia*, while individuals < 5 years of age or ≥ 40 years of age were primarily at risk for *E. coli*, *K. pneumoniae*, *P. aeruginosa*, *A. baumanni* and *E. cloacae* infection. Antibiotic resistance testing showed that MDR isolates such as MRSA and ESBL-produced *E. coli* and *K. pneumoniae* species were still a serious concern. It was particularly noteworthy that the frequency of CR-KPN isolates was increasing significantly over time.

## Abbreviations

ABA: Acinetobacter Bauman
AMK: Amikacin
AMP: Ampicillin
AMR: Antimicrobial resistance
BSI: Bloodstream infection
CARSS: China Antimicrobial Resistance Surveillance System
CHL: Chloramphenicol
CIP: Ciprofloxacin
CLI: Clindamycin
CLSI: Clinical and Laboratory Standards Institute
CP: carbapenemase-producing
CP-E/K: Carbapenemase-producing *E. coli* and *K. pneumoniae*
CR: Carbapenem-resistant
CTX: Cefotaxime
CXM: Cefuroxime
CZO: Cefazolin
ECL: *Enterobacter cloacae*
ECO: *Escherichia coli*
EFA: Enterococcus faecalis
EFM: Enterococcus faecium
ERY: Erythromycin
ESBL: Extended-spectrum β-lactamase
EuSCAPE: Carbapenemase-Producing Enterobacteriaceae in Europe
FOX: Cefoxitin
IPM: Imipenem
KP-BSIs: Bloodstream infections caused by klebsiella pneumonia
KPN: Klebsiella pneumonia
LNZ: Linezolid
LVX: Levofloxacin
MDR: Multi-drug resistant
MEM: Meropenem
MIC: Minimum inhibitory concentration
MNO: Minocycline
MRSA: Methicillin-resistant *S. aureus*
PAE: Pseudomonas aeruginosa
PEN: Penicillin
PIP: Piperacillin
PMA: Stenotrophomonas maltophilia
SAM: Ampicillin-sulbactam
SAU: *Staphylococcus aureus*
SCN: Coagulase negative staphylococci
SPN: Streptococcus pneumonia
SXT: Trimethoprim Sulfamethoxazole
TCC: Ticarcillin-Clavulanate Acid
TEC: Teicoplanin
TZP: Piperacillin-tazobactam
VAN: Vancomycin
WHO: World Health Organization
